# Why Most Clinical Research Is Not Useful

**DOI:** 10.1371/journal.pmed.1002049

**Published:** 2016-06-21

**Authors:** John P. A. Ioannidis

**Affiliations:** 1 Stanford Prevention Research Center, Department of Medicine and Department of Health Research and Policy, Stanford University School of Medicine, Palo Alto, California, United States of America; 2 Meta-Research Innovation Center at Stanford (METRICS), Stanford University, Palo Alto, California, United States of America

## Abstract

John Ioannidis argues that problem base, context placement, information gain, pragmatism, patient centeredness, value for money, feasibility, and transparency define useful clinical research. He suggests most clinical research is not useful and reform is overdue.

**Summary Points**

- Blue-sky research cannot be easily judged on the basis of practical impact, but clinical research is different and should be useful. It should make a difference for health and disease outcomes or should be undertaken with that as a realistic prospect.
- Many of the features that make clinical research useful can be identified, including those relating to problem base, context placement, information gain, pragmatism, patient centeredness, value for money, feasibility, and transparency.
- Many studies, even in the major general medical journals, do not satisfy these features, and very few studies satisfy most or all of them. Most clinical research therefore fails to be useful not because of its findings but because of its design.
- The forces driving the production and dissemination of nonuseful clinical research are largely identifiable and modifiable.
- Reform is needed. Altering our approach could easily produce more clinical research that is useful, at the same or even at a massively reduced cost.

Practicing doctors and other health care professionals will be familiar with how little of what they find in medical journals is useful. The term “clinical research” is meant to cover all types of investigation that address questions on the treatment, prevention, diagnosis/screening, or prognosis of disease or enhancement and maintenance of health. Experimental intervention studies (clinical trials) are the major design intended to answer such questions, but observational studies may also offer relevant evidence. “Useful clinical research” means that it can lead to a favorable change in decision making (when changes in benefits, harms, cost, and any other impact are considered) either by itself or when integrated with other studies and evidence in systematic reviews, meta-analyses, decision analyses, and guidelines.

There are many millions of papers of clinical research—approximately 1 million papers from clinical trials have been published to date, along with tens of thousands of systematic reviews—but most of them are not useful. Waste across medical research (clinical or other types) has been estimated as consuming 85% of the billions spent each year [1]. I have previously written about why most published research is false [2] and how to make more of it true [3]. In order to be useful, clinical research should be true, but this is not sufficient. Here I describe the key features of useful clinical research (Table 1) and the current state of affairs and suggest future prospects for improvement.

**Table 1:** Features to consider in appraising whether clinical research is useful.

Feature	Questions to Ask
Problem base	Is there a health problem that is big/important enough to fix?
Context placement	Has prior evidence been systematically assessed to inform (the need for) new studies?
Information gain	Is the proposed study large and long enough to be sufficiently informative?
Pragmatism	Does the research reflect real life? If it deviates, does this matter?
Patient centeredness	Does the research reflect top patient priorities?
Value for money	Is the research worth the money?
Feasibility	Can this research be done?
Transparency	Are methods, data, and analyses verifiable and unbiased?

Making speculative, blue-sky research more productive represents a partly intractable problem, given the unpredictability of such research, but significantly improving clinical research—and developing tools for assessing its utility or lack thereof—appears conceptually more straightforward.

## Features of Clinically Useful Research

### Problem Base

There is higher utility in solving problems with higher disease burdens. However, context is important. Solving problems with low prevalence but grave consequences for affected patients is valuable, and broadly applicable useful research may stem from studying rare conditions if the knowledge is also relevant to common conditions (e.g., discovering the importance of the proprotein convertase subtilisin-kexin type 9 [PCSK9] pathway in familial hypercholesterolemia may help develop treatments for many other patients with cardiovascular disease). Furthermore, for explosive epidemics (e.g., Ebola), one should also consider the potential burden if the epidemic gets out of control.

Conversely, clinical research confers actual disutility when disease mongering [4] creates a fictitious perception of disease burden among healthy people. In such circumstances, treated people, by definition, cannot benefit, because there is no real disease to treat.

Data show only weak or modest correlations between the amount of research done and the burden of various diseases [5,6]. Moreover, disease mongering affects multiple medical specialties [4,7,8].

### Context Placement and Information Gain

Useful clinical research procures a clinically relevant information gain [9]: it adds to what we already know. This means that, first, we need to be aware of what we already know so that new information can be placed in context [10]. Second, studies should be designed to provide sufficiently large amounts of evidence to ensure patients, clinicians, and decision makers can be confident about the magnitude and specifics of benefits and harms, and these studies should be judged based on clinical impact and their ability to change practice. Ideally, studies that are launched should be clinically useful regardless of their eventual results. If the findings of a study are expected to be clinically useful only if a particular result is obtained, there may be a pressure to either obtain that result or interpret the data as if the desired result has been obtained.

Most new research is not preceded or accompanied by systematic reviews [10,11]. Interventions are often compared to placebos or normal care, despite effective interventions having previously been demonstrated. Sample-size calculations almost always see each trial in isolation, ignoring other studies. Across PubMed, the median sample size for published randomized trials in 2006 was 36 per arm [12]. Nonvalidated surrogate outcomes lacking clinical insight [13] and composite outcomes that combine outcomes of very different clinical portent [14] are often utilized so that authors can claim that clinical studies are well powered. The value of “negative” results is rarely discussed when clinical studies are being designed.

### Pragmatism

Research inferences should be applicable to real-life circumstances. When the context of clinical research studies deviates from typical real-life circumstances, the question critical readers should ask is, to what extent do these differences invalidate the main conclusions of the study? A common misconception is that a trial population should be fully representative of the general population of all patients (for treatment) or the entire community (for prevention) to be generalizable. Randomized trials depend on consent; thus, no trial is a perfect random sample of the general population. However, treatment effects may be similar in nonparticipants, and capturing real-life circumstances is possible, regardless of the representativeness of the study sample, by utilizing pragmatic study designs.

Pragmatism has long been advocated in clinical research [15], but it is rare. Only nine industry-funded pragmatic comparative drug effectiveness trials were published between 1996 and 2010 according to a systematic review of the literature [16], while thousands of efficacy trials have been published that explore optimization of testing circumstances.

Studying treatment effects under idealized clinical trial conditions is attractive, but questions then remain over the generalizability of the findings to real-life circumstances. Observational studies (performed in the thousands) are often precariously interpreted as able to answer questions about causal treatment effects [17]. The use of routinely collected data is typically touted as being more representative of real life, but this is often not true. Most of the widely used observational studies deal with peculiar populations (e.g., nurses, physicians, or workers) and/or peculiar circumstances (e.g., patients managed in specialized health care systems or covered by specific insurance or fitting criteria for inclusion in a registry). Eventually, observational studies often substantially overestimate treatment effects [18,19].

### Patient Centeredness

Useful research is patient centered [20]. It is done to benefit patients or to preserve health and enhance wellness, not for the needs of physicians, investigators, or sponsors. Useful clinical research should be aligned with patient priorities, the utilities patients assign to different problems and outcomes, and how acceptable they find interventions over the period for which they are indicated. Proposed surrogate outcomes used in research need to closely correlate with real patient-relevant outcomes for patients in the clinic.

There is currently a heightened interest in patient-centered research, as exemplified by the Patient-Centered Outcomes Research Institute (PCORI), which was launched in 2012 in the United States to foster research relevant to patient needs [21]. Similar activities are ongoing in the United Kingdom and elsewhere. However, patients are still rarely involved in setting research priorities, despite the frequent mismatch between patient priorities and research agenda. Patients and physicians are frequently bombarded with information that tries to convince them that surrogates or other unimportant outcomes are important—such short-cuts either have commercial benefits or facilitate fast publication and academic advancement.

### Value for Money

Good value for money is an important consideration, especially in an era of limited resources, and this can be assessed with formal modeling (value of information) [22]. Different studies may require very different levels of financial investment and may differ substantially in how much we can learn from them. However, the benefits of useful clinical research more than offset the cost of performing it [23].

Most methods for calculating value for money remain theoretical constructs. Practical applications of value-of-information methods are counted in single digit numbers [24,25]. Clinical research remains extremely expensive, even though an estimated 90% of the present cost of trials could be safely eliminated [26,27]. Reducing costs by streamlining research could do more than simply allow more research to take place. It could help make research better by reducing the pressure to cut corners, which leads to studies lacking sufficient power, precision, duration, and proper outcomes to convincingly change practice.

### Feasibility

Even if all other features are met, some studies may be very difficult or practically impossible to conduct. Feasibility of research can sometimes be difficult to predict up front, and there may be unwarranted optimism among investigators and funders.

Many clinical trials are terminated because of futility. Twenty-five percent of the trials approved by six research ethics committees between 2000 and 2003 in Canada, Germany, and Switzerland were discontinued [28], and the discontinuation rate was 43% for a cohort of surgical trials registered between 2008 and 2009 [29]. For other types of research, feasibility problems are less accurately known but probably even more common.

### Transparency (Trust)

Utility decreases when research is not transparent, when study data, protocols, and other processes are not available for verification or for further use by others. Trust is also eroded when major biases occur in the design, conduct, and reporting of research.

Only 61% of trials published in clinical journals in 2010 had been registered [30], and rates are much lower for nonregulated interventions [31] (e.g., 21% and 29% for trials published in psychological or behavioral [32] and physical therapy [33] journals, respectively). Only 55/200 (28%) of journals that publish clinical trials required trial registration as of 2012 [34]. Few full protocols are registered, analysis plans are almost never prespecified, and the full study data are rarely available [35]. Trust has been eroded whenever major subversion of the evidence has been uncovered by legal proceedings [36] or reanalysis [37] with different conclusions (e.g., as in the case of neuraminidase inhibitors for influenza) [38]. Biases in the design, analysis, reporting, and interpretation remain highly prevalent [39–41].

### Other Considerations

#### Uncertainty

Some uncertainty may exist for each of the features of clinical research outlined above, even though it is less than the uncertainty inherent in blue-sky and preclinical investigation. Uncertainty also evolves over time, especially when research efforts take many years. Questions can lose their importance when circumstances change. In one of my first papers, a systematic review of zidovudine monotherapy [42], the question was extremely relevant when we started work in 1993 and still important when the paper was accepted in late 1994. However, by the time the study was published in mid-1995, the question was of no value, as new highly effective regimens had emerged: clinical utility was demolished by technological advances.

#### Other sources of evidence besides trials

Observational studies often add more confusion rather than filling the information deficits [18,19]. Meta-analyses, decision analyses, and guidelines cannot really salvage the situation based on largely useless studies and may add their own problems and biases [43–45].

#### Focusing on major journals

Some clinicians prefer to read only research published in major general medical journals (*The New England Journal of Medicine*, *The Lancet*, *BMJ*, *JAMA*, and *PLOS Medicine*). However, these journals cover a tiny minority of published clinical research. Out of the 730,447 articles labeled as “clinical trial” in PubMed as of May 26, 2016, only 18,231 were published in the major medical journals. Most of the articles that inform guidelines and clinical practice are published elsewhere. Studies in major general medical journals may do better in terms of addressing important problems, but given their visibility, they can also propagate more disease mongering than less visible journals. Clinical trials published in major medical journals are larger on average (e.g., median sample size 3,116 and 3,104, respectively, for papers published in *The Lancet* and *BMJ* in September 2007 [46]). However, the small clinical trials published in major general journals actually have more exaggerated results, on average, than equally small studies published elsewhere [47]. *The Lancet* requires routinely systematic placement of the research in context for trials, and increasingly, major journals request full protocols for published trials. Pragmatism, patient centeredness, assessments of value for money, and transparency and protection from bias remain suboptimal for most clinical research published in major journals (Table 2).

**Table 2:** How often is each utility feature satisfied in studies published in major general medical journals and across all clinical research?*

Feature	Studies Published in Major General Medical Journals	All Clinical Research
Problem base	Varies a lot	Minority
Context placement	Varies per journal (uncommon to almost always)	Uncommon
Information gain	Majority	Rare
Pragmatism	Rare	Rare
Patient centeredness	Rare/uncommon	Rare
Value for money	Unknown, rare assessments	Unknown, rare assessments
Feasibility	Almost always	Majority
Transparency	Rare/uncommon (data sharing)**, almost always (trial registration), uncommon (other study registration)	Rare/uncommon, except for trial registration (still only a minority)

*Rare: satisfied in <1% of studies; uncommon: satisfied in 1%–20% of studies; minority: satisfied in 20%–50% of studies; majority: satisfied in 50%–80% of studies; very common: satisfied in 80%–99% of studies; almost always: satisfied in >99% of studies. For supporting evidence for these estimates, see references cited in the text.

**The situation is improving in recent years for clinical trials.

### Overall Picture

Ultimately, no utility feature is met by the majority of clinical research studies, perhaps with the exception of feasibility (Table 2). Studies that meet all utility features or almost all of them are extreme rarities, even in the most highly selective journals.

## Improving the Situation

The problem of nonuseful research should not be seen as a blame game against a specific group (e.g., clinical researchers) but instead should be seen as an opportunity to improve. The challenges and the problems to solve involve not only researchers but also institutions, funding mechanisms, the industry, journals, and many other stakeholders, including patients and the public. Joint efforts by multiple stakeholders may yield solutions that are more likely to be more widely adopted and thus successful [3].

### Clinical Research Workforce and Physicians

The clinical research workforce is huge: millions of people have coauthored at least one biomedical paper, and most have done so only once [48]. Students, residents, and clinical fellows are often expected to do some research. This exposure can be interesting, but trainees are judged on their ability to rapidly produce publications, a criterion that lends itself badly to the production of the sort of large, long-term, team-performed studies often needed to inform us about health, disease, and health care. Such researchers can become exploited as low-paid or volunteer personnel [49], and an untrained, noncommitted workforce cannot produce high-quality research. Other perverse recipes in clinical research include universities and other institutions simply asking for more papers (e.g., least publishable units) instead of clinically useful papers and clinical impact not being a formal part of the publication metrics so often used to judge academic performance. Instead of trying to make a prolific researcher of every physician, training physicians in understanding research methods and evidence-based medicine may also help improve the situation by instilling healthy skepticism and critical thinking skills.

### The Industry–Regulator Dipole and Academic Partners

The industry and regulators are a closely connected dipole in licensing drugs and other products. Industry responds to regulatory requirements, and regulatory agencies increasingly act as both guardians of the common good and industry facilitators. This creates tension and ambiguity in mission. Industry should be enabled to better champion useful clinical research, with regulators matching commercial rewards to clinical utility for industry products, thus helping good companies outperform bad ones and aligning the interests of shareholders with those of patients and the public. Regulatory agencies may need to assume a more energetic role towards ensuring the conduct of large, clinically useful megatrials.

Current research funding incentivizes small studies of short duration that can be quickly performed and generate rapidly publishable results, while answering important questions may sometimes require long-term studies whose financial needs exceed the resources of most currently available funding cycles. Partnerships with patient-centered research initiatives [50] and academia can potentially solve some of the challenges of designing and implementing more pragmatic trials [51]. One should acknowledge that even for streamlined randomized trials, the cost may be substantial if multiple such trials require support by public funds. The industry may still participate by contributing funds towards a common pool of resources under public control for trials conducted by nonconflicted academic investigators. One to two percent of the sales of blockbuster drugs diverted in such a pool [52] could earmark ample funding.

### Funding Agenda for Blue-Sky, Preclinical, and Clinical Science

Discovery research without prespecified deliverables—blue-sky science—is important and requires public support. However, a lot of “basic” investigation does have anticipated deliverables, like research into developing new drug targets or new tests. This research may best be funded by industry and those standing to profit if they deliver a product that is effective. Much current public funding could move from such preclinical research to useful clinical research, especially in the many cases in which a lack of patent protection means there is no commercial reason for industry to fund studies that might nevertheless be useful in improving care. Reallocation of funds could help improve all research (basic, preclinical, and clinical) (Table 3).

**Table 3:** Funding of different types of research: Prespecified deliverables, utility, current funders, and ideal funders.

Type of Research	Prespecified Deliverables	Utility	Current Major Funder	Ideal Major Funder
Discovery “blue sky” science	No (high uncertainty by default)	Possible, but in entirely unpredictable ways, maybe decades later; very high failure rate per single idea/project explored	Public (e.g., NIH)	Public (e.g. NIH)
Applied preclinical research	Yes (uncertainty is substantial, but goals should be set)	Possible; substantial failure rate in single projects, but eventually the accumulated efforts should pay off	Public (e.g., NIH)	Entrepreneurs and industry who will profit if they deliver something that truly works; current public funding in this area should shift to clinical research instead
Clinical research	Yes (uncertainty is usually manageable, explicit goals should be set)	Yes; results should be sufficiently useful regardless of whether they are “positive” or “negative” (even if some particular results end up being more useful than others)	Industry	Public (e.g., NIH, PCORI); industry may contribute some funds to a common funding pool; regulatory agencies and universities/research institutions should safeguard the independence of research and may steer overall agenda

NIH, National Institutes of Health; PCORI, Patient-Centered Outcomes Research Institute

### Journals

Journals can be very influential is setting standards of acceptable research. External groups could also appraise the clinical utility of the papers published in journals. For example, one could track a “Journal Clinical Usefulness Factor” scoring some features mentioned above.

### Patients and Related Advocacy Groups

Patients and related advocacy groups stand to gain most by an increase in clinically useful research. These groups can influence positively the utility of research when they are savvy about science-in-the-making and protected from biased influences. Public media and related commentators of health news [53] may also help by focusing on the need to obtain clinically useful research and not compromise for less.

### Conclusion

Overall, not only are most research findings false, but, furthermore, most of the true findings are not useful. Medical interventions should and can result in huge human benefit. It makes no sense to perform clinical research without ensuring clinical utility. Reform and improvement are overdue.

## Abbreviations

NIH: National Institutes of Health
PCORI: Patient-Centered Outcomes Research Institute
PCSK9: proprotein convertase subtilisin-kexin type 9
